# In Search of Antioxidant Peptides from Porcine Liver Hydrolysates Using Analytical and Peptidomic Approach

**DOI:** 10.3390/antiox11010027

**Published:** 2021-12-24

**Authors:** María López-Pedrouso, José M. Lorenzo, Paula Borrajo, Daniel Franco

**Affiliations:** 1 Departamento de Zooloxía, Xenética e Antropoloxía Física, Universidade de Santiago de Compostela, 15872 Santiago de Compostela, Spain; mariadolores.lopez@usc.es; 2 Centro Tecnolóxico da Carne de Galicia, Rúa Galicia No. 4, Parque Tecnolóxico de Galicia, San Cibrao das Viñas, 32900 Ourense, Spain; jmlorenzo@ceteca.net (J.M.L.); paulaborrajo@ceteca.net (P.B.); 3 Área de Tecnoloxía dos Alimentos, Facultade de Ciencias, Universidade de Vigo, 32004 Ourense, Spain

**Keywords:** amino acid profile, alcalase, bromelain, flavourzyme, papain, pork by-products

## Abstract

The search for antioxidant peptides as health-promoting agents is of great scientific interest for their biotechnological applications. Thus, the main goal of this study was to identify antioxidant peptides from pork liver using alcalase, bromelain, flavourzyme, and papain enzymes. All liver hydrolysates proved to be of adequate quality regarding the ratio EAA/NEAA, particularly flavourzyme hydrolysates. The peptidomic profiles were significantly different for each enzyme and their characterizations were performed, resulting in forty-four differentially abundant peptides among the four treatments. Porcine liver hydrolysates from alcalase and bromelain are demonstrated to have the most antioxidant capacity. On the other hand, hydrophobic amino acid residues (serine, threonine, histidine and aspartic acid) might be reducing the hydrolysates antioxidant capacity. Seventeen peptides from collagen, albumin, globin domain-containing protein, cytochrome β, fructose-bisphosphate aldolase, dihydropyrimidinase, argininosuccinate synthase, and ATP synthase seem to be antioxidant. Further studies are necessary to isolate these peptides and test them in in vivo experiments.

## 1. Introduction

Novel antioxidant peptides play an increasingly important role in biotechnological applications; consequently, the nutraceutical, functional food, and cosmetic sectors are paying more and more attention to them. In this respect, industrial processing wastes with high content of protein provide an opportunity for preparing hydrolysates with antihypertensive, antioxidant, antimicrobial, antidiabetic, anticancer, and other bio-activities [1]. Moreover, the field of peptidomics as a part of proteomic is becoming more crucial in search of bioactive peptides. Indeed, from 2015 to 2020, a total of 80 original papers were published according to the Scopus database, using the keywords “peptidomic” and “biopeptide”. More specifically, a systematic search of the literature using the keywords most important of this paper (“bioactive peptides” and “pork liver”) resulted in only five original papers. Therefore, great development of technical capacity is being carried out in recent years, mainly performed by liquid chromatography to separate the peptides followed by mass spectrometry in tandem to identify them. Ultrafiltration, hydrophobic-high-performance liquid chromatography (HPLC), ion-exchange HPLC, and capillary electrophoresis in most cases coupled to electrospray ionization are the main analytical instruments used. Finally, the protein identification is carried out by Q-TOF or MALDI-TOF/TOF instrumentation as a mass spectrometer [2,3].

Protein-rich by-products of animal origin (fish, seafood, milk, and meat products) are valuable sources of these bioactive compounds. In addition, recycling and reusing these animal wastes lead us to reach a more sustainable industry as a second objective [4]. To achieve this purpose, the preferred method to obtain a peptide mixture is enzymatic hydrolysis. Usually, food proteins are hydrolyzed by peptidases and, in most cases, microbial peptidases cause different peptide patterns (Toldrá, Reig, Aristoy, and Mora, 2018), but there are other enzymes from fruit and vegetables. In any case, the enzymatic hydrolysis requires optimization concerning the reaction conditions including the selection of the enzyme, reaction time, and temperature for each enzyme. Regarding pork liver hydrolysates, various enzymes including alcalase, bromelain, flavourzyme, and papain have been employed. In this sense, the optimization of enzymatic reaction was carried out to increase the degree of hydrolysis resulting in 27.5% with alcalase 2.4L™ and novo Pro-D™ [5].

Within the meat industry, in the last years, there has been a demanding interest in pork liver which is an adequate source of protein (18.54%) with a low-fat percentage (3.38%) and phospholipids, with great benefits for human health [6]. Furthermore, the amino acid profile is comprised of aromatic (tyrosine and phenylalanine) and hydrophobic (leucine, valine, and isoleucine) amino acids that show high antioxidant capacity [7]. Other abundant proteins from animal sources as keratin and collagen seem to be inactive molecules because of their high stable protein structures [8]. In this regard, smaller antioxidant peptides (4–16 amino acids with a molecular weight of 400–2000 Da) have also been found in other animal tissue [9].

Since studies linked to the antioxidant effect of pork liver-derived peptides are still limited, this study aimed to evaluate the effect of several enzymes on pork liver in the generation of antioxidant peptides. To this end, a high number of antioxidant peptides needs to be identified using different techniques and, further, to isolate them for validation of the results.

## 2. Materials and Methods

### 2.1. Preparation of Porcine Liver Hydrolysate

The fresh porcine livers (*n* = 6) were provided by a local meat retailer (Cárnicas M. Boo, Ourense, Spain). The connective tissues and external fat were removed from the porcine liver before they were chopped and frozen at −20 °C till further use. Enzymatic hydrolysis was carried out using bioprotease LA 660 (Alcalase), bromelain 2000 U/g, and Papain 6000 USP provided by Biocon (Barcelona, Spain), meanwhile flavourenzyme^®^ 1000 L was supplied by Novozymes (Bagsværd, Denmark). The treatments for each enzyme were performed completely separately. Homogenization of samples was performed by mixing with ice (1:1 liver/ice) in a cutter machine (Talsa K3, Valencia, Spain) for 30 min before adding the enzyme. The enzymatic hydrolysis was performed at optimum pH and temperature described for each enzyme: Alcalase (50 °C, pH = 8), bromelain (40 °C, pH = 6), flavourenzyme^®^ (50 °C, pH = 5.5), and papain (37 °C, pH = 6). An enzyme-substrate ratio of 1:100 (*w*/*w*) was employed in all incubations for seven hours under agitation in an orbital shaker incubator (125 rpm). During the hydrolysis, periodic adjustments of pH were performed by employing NaOH or HCl 1N. Finally, hydrolysates were heated at 95 °C for 3 min to deactivate the enzymes and, afterwards, liver homogenates were cooled in an ice bath. Hydrolysates were centrifuged (Allegra X-22R Centrifuge, Beckman Coulter, Barcelona, Spain) at 4000× *g* for 10 min. The purified extracts were frozen at −80 °C until analysis. For each enzymatic treatment, hydrolysis was conducted in triplicate.

### 2.2. Free Amino Acid Profile

The extraction of free amino acids was carried out as follows: hydrolysates extract (0.5 g) was homogenized with 25 mL of HCl 0.1 M for 8 min employing a disperser (Ika, T 25 digital Ultra-Turrax^®^, Staufen, Germany). Afterwards, it was cooled and centrifuged at 5000× *g* for 20 min and 200 µL of the supernatant was mixed with 800 µL of acetonitrile to precipitate possible unhydrolyzed proteins. Finally, centrifugation at 5000× *g* for 5 min was performed and an aliquot was frozen at −20 °C and stored until analysis. Derivatization and separation by HPLC were carried out according to Franco and Lorenzo [10]. Free amino acids were identified by retention time using an amino acid standard and expressed in mg/100 g of pork liver.

### 2.3. Antioxidant Activity

#### 2.3.1. DPPH Radical Scavenging Activity

The DPPH assay was performed following Brand-Williams et al. [11] with slight modifications. A sample of 100 μL was mixture with 3900 μL of DPPH solution (60 μM in methanol) and incubated for 10 min at 37 °C. Absorbance was measured at 515 nm (Shimadzu spectrophotometer, Kyoto, Japan). Trolox reactive was the standard used and results were expressed as μg Trolox equivalents (TE)/g sample.

#### 2.3.2. ABTS Radical Scavenging Activity

ABTS Radical Cation Decolorization was performed following Re et al. [12] with slight modifications. ABTS was prepared by mixing 7 mM ABTS stock solution with 2.45 mM potassium persulfate and maintaining the mixture in the dark at room temperature for 12–16 h before its utilization. Before use, the ABTS stock solution was diluted with distilled water to achieve an absorbance of 0.70 at 734 nm, being equilibrated at 30 °C. The solution (980 mL) was added to an aliquot of 20 mL of each hydrolysate/standard. Absorbance was measured after 10 min in the darkness. Ascorbic acid was the standard used and results were expressed as mg ascorbic acid/100 g sample.

#### 2.3.3. Ferric Reducing Antioxidant Power Assay (FRAP)

The FRAP test was carried out following Benzie and Strain [13], with minor changes. FRAP reagent was freshly prepared from 0.3 M acetate buffer (pH 3.6), 10 mM 2,4,6-tripyridyl-s-triazine in 40 mM HCl and 20 mM FeCl_3_:6H_2_O in the ratio of 10:1:1 (*v*:*v*:*v*). An aliquot of 900 mL of FRAP solution was mixed with 30 μL of properly diluted samples and 90 μL of distilled water. After incubation for 20 min at 37 °C in the darkness, the absorbance was determined at 593 nm. The FeSO_4_ was the standard used and results were expressed as μmol Fe^+2^/100 g sample.

#### 2.3.4. Oxygen Radical Absorbance Capacity Assay (ORAC)

The ORAC test was performed following Huan et al. [14] with minor modifications. The reaction was carried out in 75 mM phosphate buffer (pH 7.4), being 200 µL of the final volume of the reaction mixture. Twenty-five µL of dilute sample and 150 µL of 0.8 µM fluorescein (oxidizable substrate) were added into the internal wells of a black 96-well microplate (Biotek, Synergy H1, Winooski, VT, USA) and which was immediately incubated at 37 °C for 30 min in the own fluorescence instrument. Afterwards, 25 µL of 2,2-azobis (2-methylpropionamidine) dihydrochloride 184 mM solution were added rapidly to each well to begin the reaction in the microplate. The fluorescence was measured with excitation and emission filters of 485 nm and 528 nm, respectively. The phosphate buffer and Trolox were used as the blank and standard reference, respectively. The results were estimated based on the differences of areas under the curves of fluorescence decay of the fluorescein between the blank and the sample and expressed as mg Trolox Equivalent (TE)/g sample.

### 2.4. Peptidomic Analysis

#### 2.4.1. Identification of Peptides by Liquid Chromatography and Tandem Mass Spectrometry (LC-MS/MS) Analysis

A peptide mixture of 3 µL was loaded onto a trap column (3µ C18-CL 120 Ᾰ, 350 μm × 0.5 mm; Eksigent, AB Sciex, Alcobendas, Madrid) and desalted with 0.1% TFA at 5 µL/min for 5 min. Afterwards, the peptides were loaded onto the column (3µ C18-CL 120 Ᾰ, 0.075 × 150 mm; Eksigent, AB Sciex, Alcobendas, Madrid) equilibrated in 5% acetonitrile 0.1% FA (formic acid). Elution was done with a linear gradient from 7% to 45% B in A for 20 min. (A: 0.1% FA; B: ACN, 0.1% FA) at a flow rate of 300 nL/min. Peptides were identified in a mass spectrometer nanoESI qQTOF (6600 plus TripleTOF, SCIEX, Framingham, MA, USA) in a data-dependent mode. Samples were ionized in a Source Type: Optiflow < 1 µL Nano applying 3.0 kV to the spray emitter at 200 °C. Survey MS1 scans were acquired from 350–1400 *m*/*z* for 250 ms. The quadrupole resolution was set to ‘LOW’ for MS2 experiments, which were acquired 100–1500 *m*/*z* for 25 ms in ‘high sensitivity’ mode using the following switch criteria: charge: +1 to +4; minimum intensity; 100 counts per second. Up to 50 ions were selected for fragmentation after each survey scan. Dynamic exclusion was set to 15 s. The system sensitivity was controlled by analyzing 500 ng of K562 trypsin digestion. In these conditions, 2260 proteins were identified (FDR < 1%) in 45 min gradient.

ProteinPilot v 5.0. (SCIEX) default parameters were employed to generate peak lists directly from 6600 plus TripleTOF wiff files. The Paragon algorithm [15] of ProteinPilot v 5.0 was used to search the Uniprot mammals database with the following parameters: none enzyme specificity, taxonomy restricted to pig, and the search effort set to rapid.

#### 2.4.2. Label-Free Relative Quantitative Analysis by Mass Spectrometry

The quantification of peptides was performed according to the label-free methodology described by [2]. This approach is based on the measurement of relative ion intensities of extracted ion chromatograms (XICs) to determine the ratios for individual peptides, employing three replicates per digested hydrolysate. Peptides were quantified using PeakView v1.1 software (AB Sciex, Framingham, MA, USA) and analyzed with Marker View v1.3 software (AB Sciex, Framingham, MA, USA). The protein grouping was carried out by the Pro group algorithm. A protein group in a Pro Group Report is a set of proteins that share some physical evidence. Unlike sequence alignment analyses where full-length theoretical sequences are compared, the formation of protein groups in Pro Group is guided entirely by observed peptides only. As observed peptides were identified from experimentally acquired spectra, the grouping can be guided by spectra usage. Then, unobserved regions of protein sequence play no role in explaining the data.

### 2.5. Statistical Analysis

Statistical analysis was performed employing the IBM SPSS Statistics 23.0 program (IBM Corporation, Somers, NY, USA). An ANOVA was applied to evaluate the effect of each enzyme treatment on the antioxidant activity of the hydrolysate. The least-square means (LSM) of the four treatments were separated using Duncan’s post hoc test. All statistical tests of LSM were performed for a significance level of *p* < 0.05. Correlations among antioxidants tests (*p* < 0.01) and identified and quantified amino acids and peptides were determined employing Pearson’s linear correlation coefficient. To better understand the relationships among the different effects of the enzymes over hydrolysates antioxidant activity, a cluster analysis based on the unweighted pair group method with arithmetic mean (UPGMA) dendrogram was performed using XLSTAT 2021.3.1 (Addinsoft, Paris, France).

## 3. Results

### 3.1. Characterization of Porcine Liver Hydrolysates by Enzymatic Reaction

In the present study, the enzymatic hydrolysis was carried out using four enzymes, alcalase, bromelain, flavourzyme, and papain, for 7 h to reach the most antioxidant activity.

#### 3.1.1. Amino Acid Composition of Porcine Liver Hydrolysates

The free amino acids (FAA) profiles which also provide a rough idea of the hydrolysis process are shown in Table 1. The highest amount of FAA was achieved using flavourzyme (3065.13 mg/100 g liver) and the lowest in papain hydrolysates (1285.73 mg/100 g liver), reaching statistical differences (*p* < 0.05). On the contrary, between hydrolysates from alcalase (1572.38 mg/100 g liver) and bromelain (1904.05 mg/100 g liver), no significant differences (*p* > 0.05) were found on FAA total content. All liver hydrolysates FAA profiles from the four treatments were characterized by a high level of leucine, lysine, and valine in the essential fraction. Indeed, leucine ranged from 152.95 to 353.05 mg/100 g protein after hydrolysis performed by papain and flavourzyme, respectively. In the case of valine, the lower value (77.80 mg/100 g protein) and the higher value (220.51 mg/100 g protein) were also produced by flavourzyme and papain, respectively.

#### 3.1.2. Peptide Composition of Porcine Liver Hydrolysates

**Figure 1:**
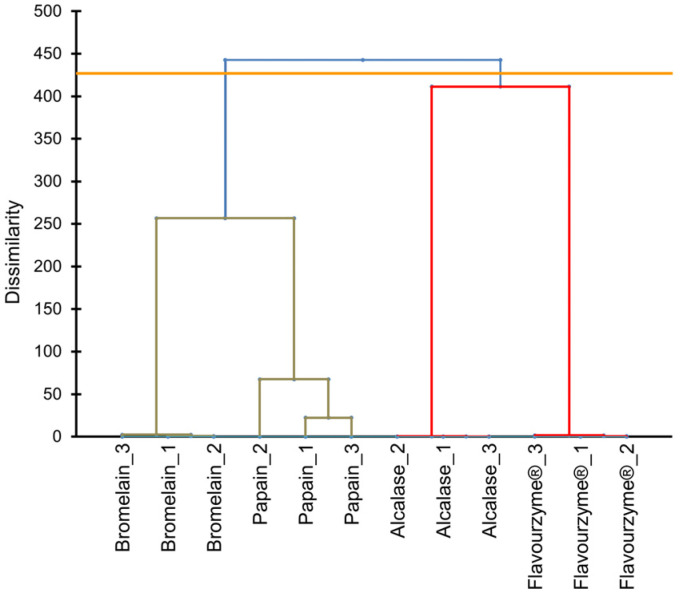
Cluster analysis using the unweighted pair group method with arithmetic mean (UPGMA) dendrogram based on peptides quantifications provided by the action of alcalase, bromelain, papain, and flavouryme^®^ on pork liver.

The peptidomic profiles in pork liver hydrolysates were inferred using the quantifications of LC-MS/MS. After enzymatic treatments, the peptides from pork liver were identified and quantified by LC-MS/MS. For the cluster analysis, only data from peptides identified with differential abundance (44 peptides) were considered in the UPGMA dendrogram (Figure 1). As can be observed, hydrolysates of enzymatic treatments (alcalase, bromelain, flavourenzyme, and papain) were grouped into four groups. This finding demonstrated that the peptide profile is different and distinctive for each enzymatic treatment.

### 3.2. Antioxidant Capacity of Porcine Liver Hydrolysates

**Figure 2:**
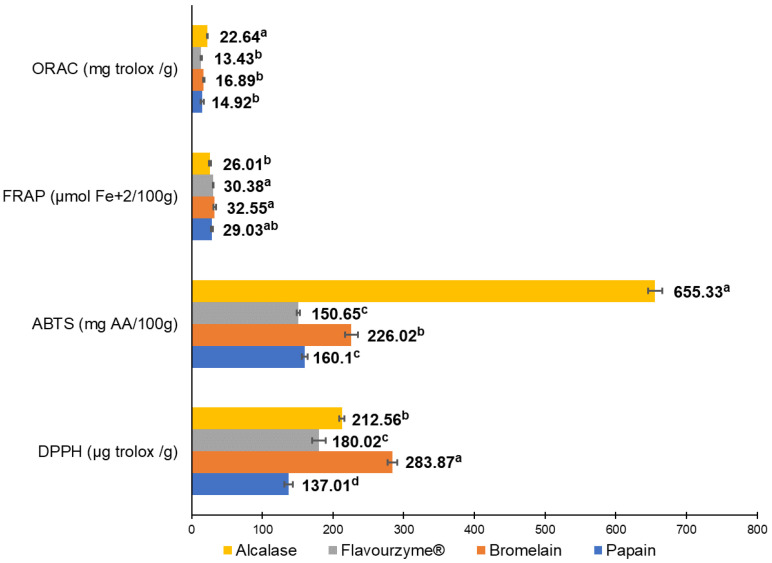
Antioxidant in vitro tests to evaluate antioxidant capacity from the four different pork liver hydrolysates produced by alcalase, bromelain, papain, and flavouryme. a to d upper letters indicate significant differences at *p* < 0.05 level using Duncan test.

The antioxidant capacity of pork liver hydrolysates was measured using DPPH, ABTS, FRAP, and ORAC as shown in Figure 2. Hydrolysates from alcalase and bromelain resulted in the most antioxidant capacity. Alcalase enzyme generated the highest antioxidant activity measured by ORAC (22.64 mg Trolox/g) and ABTS (655.63 mg AA/100 g); meanwhile, the bromelain enzyme showed the greatest antioxidant values by FRAP (32.55 µmol Fe^+2^/100 g) and DPPH (283.87 µg Trolox/g).

#### 3.2.1. Antioxidant Effect of Free Amino Acids from Porcine Liver Hydrolysates

The antioxidant activity of these hydrolysates may largely be due to FAA. As shown in Table 2 the FAA involved in antioxidant activity have different chemical properties in their side chains as polar uncharged (serine and threonine), polar charged (histidine and aspartic acid), and apolar (proline). Furthermore, the polar FAAs decreases the antioxidant capacity (negative correlation) in contrast to proline as an apolar FAA (positive correlation). It has been demonstrated that FAAs produce different antioxidant activities depending on the properties of their side residues. Specifically, cysteine, methionine, tryptophan, tyrosine, and histidine have proved to have higher antioxidant capacity than other amino acids because they are relatively easily oxidized [16]. However, neither of these FAA significantly improved the antioxidant activity of hydrolysates in our study.

#### 3.2.2. Peptides of Porcine Liver Hydrolysates with Antioxidant Capacity

From the peptides quantified with significant differences among the four enzymatic treatments (Table 3), correlation with antioxidant capacity using ORAC, FRAP, ABTS, and DPPH tests were analyzed. From this initial set of peptides, seventeen peptides showed a significant correlation (*p* ≤ 0.05) with a coefficient correlation higher than 0.5 (Table 4).

Derived peptides of collagen resulted in a large influence on antioxidant capacity mainly correlated with DPPH both positively and negatively. The peptide SVGPVGPAGPI and SP[Oxi]GPDGKTGPP[Oxi]GPAG produced by the action of alcalase and bromelain were positively correlated with the antioxidant test of DPPH (r = 0.833 and 0.850, *p* < 0.01; respectively). On the contrary, GSP[Oxi]GPSGSP[Oxi]GQRGEP[Oxi]GP and GAP[Oxi]GDKGETGPSGPAGPT by hydrolyzation with papain were negatively correlated (r = −0.777 and −0.843, *p* < 0.05 and 0.01; respectively).

It should be noted that three peptides from albumin also resulted in antioxidant ability correlated with ABTS and ORAC assays. Thus, the peptides NDNPDIPKLKPDPV and DNPDIPKLKPDPVAL produced by alcalase action was correlated with ABTS (r = 0.917 and 0.990, *p* < 0.01; respectively) and ORAC (r = 0.729 and 0.788, *p* < 0.05; respectively). Unlike others, the peptide DFQEDEQKFW hydrolyzed by bromelain was correlated with DPPH (r = 0.836, *p* < 0.05).

Peptides with antioxidant capacity, from iron proteins including cytochrome B and haemoglobin, were also detected. As other peptides produced by alcalase, LVLMILVL (cytochrome B) was strongly correlated with ABTS and ORAC (r = 0.983 and 0.765, *p* < 0.01 and 0.05; respectively). The peptide of SDGLKHLDNLK (haemoglobin) was related to the DPPH test (r = 0.753, *p* < 0.05), as with other peptides of the bromelain enzyme.

Other peptides from the proteins related to metabolic pathways such as fructose-bisphosphate aldolase, dihydropyrimidinase, and arginosuccinate synthase were also particularly correlated with ABTS and ORAC. These peptides of metabolic proteins were particularly generated by alcalase and correlated with antioxidant capacity using ABTS and ORAC. Overall, the main candidates of antioxidant peptides from porcine liver hydrolysates resulting from alcalase action were particularly sensitive to ORAC and ABTS. Additionally, bromelain action appears to increase antioxidant peptides measured by the DPPH test.

## 4. Discussion

### 4.1. Characterization of Porcine Liver Hydrolysates by Enzymatic Reactions

To improve the sustainability of the meat industry, pork liver should be processed taking advantage of its nutritional qualities. According to [17], the porcine liver protein content is of great importance because it has the highest level of protein among the pork by-products (heart, lung, stomach, intestine, spleen, uterus, and pancreas).

Pork liver hydrolysates had a high content of leucine, lysine, and valine in the essential fraction. The branched-chain amino acids (leucine and valine) along with isoleucine are essential as precursors of glutamine and alanine with an important role in the protein synthesis with health benefits for young and elderly persons. A person of about 70 Kg should consume 2730 mg of leucine daily and the most common way is via commercial supplements based on whey protein (2.0–3.0 g leucine) [18,19]. Our data indicated that leucine, lysine, and valine as FAA were higher in liver hydrolysates from flavourzyme, resulting in these amino acids being more available. Furthermore, the highest ratio of EAA/NEAA was achieved with flavourzyme (1.16), indicating that flavourzyme hydrolysates are of higher quality in terms of free leucine and valine, as well as with respect to the nutritional index between essential and non-essential amino acid fractions. However, a major problem with pork liver is due to its sensory attributes (mainly its undesirable odor). This fact implies a low economic value, becoming a suitable tissue to extract bioactive peptides [20].

Regarding peptide composition, mixtures of peptides with sequences below 16 amino acids seem to comprise a good opportunity to search peptides with high biological activity [9]. The analysis of peptidomic pattern for the porcine liver hydrolysates from alcalase, bromelain, flavourzyme, and papain using the peptide quantification was carried out. Thus, fifty-seven peptides were identified and, among them, forty-four were significantly different in the porcine liver hydrolysates as shown in Table 2. Considering that the antioxidant effect could be correlated with the peptide concentration, only those peptides with a high concentration were taken into account.

As can be inferred from Figure 1, protein hydrolysis through bromelain and papain were quite similar, resulting in a close peptide mixture. This fact could be explained as a result of the vegetable origin of both enzymes as they have common peptidase activity. Papain enzyme is extracted from the unripe (immature and green) Carica papaya L. species whereas bromelain comes from core fruit and stem of Bromeliaceae or pineapple family, mainly from Ananascomosus Merr., sp. Bromelain, and papain enzymes are widely used to decrease the toughness of meat and fish tissues during the ageing process [21]. On the contrary, alcalase and flavourzyme are enzymes derived from microorganisms with a high degree of hydrolysis, which also were used to generate antioxidant peptides [22]. These different origins among the enzymes could be a possible explanation for the large difference in peptides rupture pattern displayed in the UPGMA dendrogram.

### 4.2. Antioxidant Capacity of Porcine Liver Hydrolysates

Different assays are used to measure the antioxidant capacity of foods based on describing the ability of redox molecules to scavenge free radicals. Among these, ORAC is considered the most relevant method for the assessment of radicals from biological origin [23]. However, each assay used to estimate antioxidant capacity should be considered depending on the nature of the antioxidant compound [24]. Consequently, in this study, the antioxidant capacity of the pork hydrolysates should be analyzed as a whole.

The antioxidant capacity of hydrolysates increased with the level of proteolysis (data not shown) suggesting that the bioactivity of complex mixture is largely due to peptides which are released. On the other hand, it was proved that proteins and peptides from meats products have high antioxidant capacity due to their ability to scavenge free radicals and chelate pro-oxidative metals. For instance, [25] reported higher antioxidant capacity in samples of dry-cured ham than those resulting from fish (sardine and hake) or vegetal products (orange juice) using H-ORACFL extraction procedure. These endogenous antioxidants have a beneficial effect on meat oxidation and are affected by the pig genotype [26], among other factors from farm to fork.

Antioxidant peptides composed of 3–6 amino acids (lower than 1 kDa) and hydrophilic amino acids are considered a key factor to improve the scavenging of radicals [27]. In addition to the abovementioned, antioxidant peptides with a hydrophilic group could improve their solubility and consequently their bioavailability [16]. In the present study, the high content of hydrophobic amino acids residues (serine, threonine, histidine, and aspartic acid) from peptides lowered the solubility and reduced their antioxidant capacity. Thus, it might suggest that each enzymatic reaction acts differentially, provoking differences in the antioxidant activity of the hydrolysates.

A relevant antioxidant capacity was detected concerning peptides from collagen and albumin. Collagen is a connective tissue protein mainly composed of hydroxyproline (12.5%), as well as proline and glycine (45–50%). It has been demonstrated that peptides from porcine collagen are a good source of natural antioxidative peptides measured by DPPH [28]. The collagen and gelatin are often extracted from the skin and bones of pigs and cows as well as fishery by-products. Thus, enzymatic hydrolysis of this protein is used to obtain the bioactive peptides as health-promoting agents [29]. Albumin is highlighted by antioxidant capacity in plasma and with high solubility. In regards to its antioxidant activity, this protein has the great ability to bind other molecules including metal ions, fatty acids, drugs, and hormones [30]. In previous studies, hydrolysates of porcine plasma obtained by alcalase treatment also showed the best antioxidant ability with peptide fractions smaller than 3 kDa even better than globulin [31].

The antioxidant capacity of iron proteins such as cytochrome B and haemoglobin may be explained by the fact that both can change their oxidation state, provoking conformational changes due to their cellular functions. Cytochrome B is a mitochondrial protein involved in the electron transport chain; meanwhile, haemoglobin is responsible for oxygen carrying through heme groups [32]. A peptide from glutathione transferase was detected and correlated with antioxidant capacity of the mixture. This is not surprising because this enzyme has the capacity to conjugate glutathione with compounds containing electrophilic centers, considerably increasing its antioxidant capacity. This enzyme is ubiquitous and plays a key role in detoxification processes [33].

## 5. Conclusions

The porcine liver is a suitable source of protein that can be easily hydrolyzed by enzymatic reactions giving rise to bioactive peptides. The action of alcalase, bromelain, flavourzyme, and papain proved to be very different concerning the resulting mixture of peptides and free amino acids. From a healthy point of view, liver hydrolysates generated by flavourzyme resulted in the highest amino acid nutritional index, providing the greatest amounts of lysine, valine and leucine, which would be beneficial for young and elderly individuals. However, alcalase and bromelain hydrolysates showed higher antioxidant capacity. Most FAA decreased the antioxidant capacity, except proline; consequently, an excessive protein degradation might not lead us to better results in terms of antioxidant activity. Porcine liver hydrolysates by alcalase provided most of the antioxidant peptides measured by ORAC and ABTS; meanwhile, those released by bromelain increased the antioxidant capacity measured by DPPH.

Seventeen peptides from collagen, albumin, globin domain-containing protein, cytochrome β, fructose-bisphosphate aldolase, dihydropyrimidinase, argininosuccinate synthase, and ATP synthase are candidate antioxidant peptides. Therefore, our findings provided potential antioxidant peptides from liver hydrolysates. Bioactive peptides production from pork livers was clearly demonstrated as tenable for commercial applications. Further studies are necessary to ensure the bioavailability of these the results with in vivo experiments.

## Figures and Tables

**Table 1:** Free amino acid profile of liver (mg/100 g liver) from hydrolysis treatment with different enzymes (alcalase, bromelain, flavourzyme, and papain).

AA	Alcalase	Bromelain	Flavourzyme	Papain	SEM	*p*-Value
**Essential**						
His	55.28 ^d^	137.76 ^b^	212.15 ^a^	89.31 ^c^	12.88	<0.0001
Iso	88.10 ^b^	66.38 ^c^	149.88 ^a^	65.11 ^c^	7.70	<0.0001
Leu	192.40 ^bc^	221.65 ^b^	353.05 ^a^	152.95 ^c^	17.40	<0.0001
Lys	189.90 ^b^	213.60 ^ab^	239.41 ^a^	74.83 ^c^	14.94	<0.0001
Met	93.21 ^b^	79.38 ^b^	142.68 ^a^	52.50 ^c^	7.33	<0.0001
Phe	83.01 ^b^	69.30 ^b^	189.71 ^a^	63.65 ^b^	11.40	<0.0001
Thr	1.08 ^d^	68.26 ^b^	140.46 ^a^	29.40 ^c^	11.05	<0.0001
Val	111.65 ^b^	79.31 ^c^	220.51 ^a^	77.80 ^c^	12.89	<0.0001
Total EAA	814.65 ^b^	935.66 ^b^	1647.88 ^a^	605.56 ^c^	87.86	<0.0001
**Non-Essential**						
Arg *	22.36 ^c^	81.88 ^b^	192.26 ^a^	8.71 ^c^	15.43	<0.0001
Ala	176.50 ^a^	124.43 ^bc^	151.41 ^ab^	98.75 ^c^	9.19	0.008
Asp	7.21 ^c^	37.96 ^b^	98.01 ^a^	38.65 ^b^	7.22	<0.0001
Cis	153.51 ^b^	37.96 ^c^	204.36 ^a^	25.11 ^c^	16.32	<0.0001
Glu	127.08 ^b^	59.40 ^c^	234.11 ^a^	137.01 ^b^	13.90	<0.0001
Gli	130.63 ^b^	161.50 ^a^	131.13 ^b^	125.88 ^b^	4.04	0.01
Pro	22.35 ^c^	229.35 ^a^	53.01 ^b^	60.55 ^b^	17.51	<0.0001
Ser	10.63 ^d^	148.00 ^b^	234.58 ^a^	93.05 ^c^	17.58	<0.0001
Tau *	16.25 ^d^	23.46 ^c^	31.40 ^b^	42.63 ^a^	2.19	<0.0001
Tyr	91.18 ^a^	64.36 ^b^	86.93 ^a^	49.80 ^c^	4.07	<0.0001
Total NEAA	757.73 ^c^	968.38 ^b^	1417.25 ^a^	680.16 ^c^	66.57	<0.0001
Total FAA	1572.38 ^bc^	1904.05 ^b^	3065.13 ^a^	1285.73 ^c^	153.79	<0.0001
Ratio EAA/NEAA	1.07 ^b^	0.96 ^c^	1.16 ^a^	0.88 ^d^	0.02	<0.0001

* Arginine and taurine are considered as semi-essential amino acids; EAA = essential amino acids; NEAA = non-essential amino acids; AA = amino acids; SEM = standard error of mean; a to d upper letters indicate significant differences at *p* < 0.05 level using Duncan test.

**Table 2:** Correlations between free amino acids and in vitro antioxidant test.

Aminoacid	ABTS	DPPH	FRAP	ORAC
His	−0.654			−0.542
Thr	−0.662			−0.559
Asp	−0.687			−0.893
Pro		0.704	0.541	
Ser	−0.770			−0.622
Tau	−0.727	−0.666		−0.591

Only those correlations significant (*p* < 0.01) in at least two tests and with a correlation coefficient higher than 0.5 are shown.

**Table 3:** Pork liver peptides identified and quantified by LC-MS/MS from hydrolysis treatment with different enzymes (alcalase, bromelain, flavourzyme, and papain).

Peptide Sequence	Protein of Origin	Gen (Uniprot ID)	Alcalase	Bromelain	Flavourzyme	Papain	SEM	*p*-Value
GVRGPNGDSGRP[Oxi]GEP[Oxi]G	Fibrillar collagen NC1 domain-containing protein	COL1A2	46,984 ^a^	237,462 ^b^	**655,298** ^**c**^	12,167 ^a^	97.120	<0.001
GSP[Oxi]GPSGSP[Oxi]GQRGEP[Oxi]GPQ	Collagen type III alpha 1 chain	COL3A1	11,730 ^a^	13,765 ^a^	**232,011** ^**b**^	22,998 ^a^	35.482	<0.001
TDPDAPSRKDPKYR	UP	PEBP1	266,420 ^c^	**322,481** ^**d**^	94,672 ^b^	10,957 ^a^	48.467	0.002
SP[Oxi]GPDGKTGPP[Oxi]GPAG	Collagen alpha-1(I) chain preproprotein	COL1A1	40,343 ^a^	**355,355** ^**b**^	45,816 ^a^	33,045 ^a^	51.950	<0.001
GSP[Oxi]GPSGSP[Oxi]GQRGEP[Oxi]GP	Collagen type III alpha 1 chain	COL3A1	27,410 ^a^	14,226 ^a^	198,625 ^b^	**277,532** ^**c**^	45.123	0.024
GASGPAGPRGPP[Oxi]GSAGAP[Oxi]GKDG	Collagen alpha-1(I) chain preproprotein	COL1A1	5415 ^a^	95,886 ^b^	**278,639** ^**c**^	107,394 ^b^	39.555	0.022
VLSAADKANVK	GLOBIN domain-containing protein	LOC110259958	7566 ^a^	12,638 ^a^	10,862 ^a^	**809,312** ^**b**^	142.092	0.042
AP[Oxi]GDKGETGPSGPAGPTG	Collagen alpha-1(I) chain preproprotein	COL1A1	443 ^a^	8189 ^a^	151,220 ^b^	**463,662** ^**c**^	73475	0.009
GKDGEAGAQGPP[Oxi]GPA	Collagen alpha-1(I) chain preproprotein	COL1A1	32,253 ^a^	10,023 ^a^	**833,315** ^**b**^	77,903 ^a^	130.966	<0.001
GVQGPP[Oxi]GPAGEEGKRG	Collagen alpha-1(I) chain preproprotein	COL1A1	9582 ^a^	19,649 ^a^	**595,576** ^**b**^	4043 ^a^	95.797	<0.001
RKPPTDEESLEK	Glutathione transferase	GSTO1	**372,448** ^**c**^	6332 ^a^	29,252 ^a^	122,768 ^b^	56.928	0.009
M[DTM]GDSRDPASDQMK	Catalase	CAT	15,852	203,806	934	428,309	72.553	0.061
GHQGAVGSP[Oxi]GPAGP	Collagen type III alpha 1 chain	COL3A1	66,649	199,720	7523	375,109	59.011	0.058
GASGPAGPRGPP[Oxi]GSA	Collagen alpha-1(I) chain preproprotein	COL1A1	9990 ^a^	17,658 ^a^	**308,010** ^**c**^	52,706 ^b^	46.659	<0.001
GPVGPSGPP[Oxi]GKDGASG	Collagen type III alpha 1 chain	COL3A1	31,327 ^a^	25,684 ^a^	14,649 ^a^	**212,882** ^**b**^	31.988	0.007
GAP[Oxi]GDKGETGPSGPAGPT	Collagen alpha-1(I) chain preproprotein	COL1A1	71,644 ^a^	8189 ^a^	267,506 ^b^	**455,180** ^**c**^	69.021	0.011
SGPAGPRGPP[Oxi]GSA	Collagen alpha-1(I) chain preproprotein	COL1A1	429,583	6914	173,859	116,358	64.360	0.051
GLP[Oxi]GTSGPP[Oxi]GENGKP[Oxi]GEP[Oxi]GPK	Collagen type III alpha 1 chain	COL3A1	40,928	588,757	285,781	156,852	126.713	0.560
GSP[Oxi]GERGEVGPAGPNG	Fibrillar collagen NC1 domain-containing protein	COL1A2	4779 ^a^	11,815 ^a^	**509,034** ^**b**^	12,027 ^a^	81.786	<0.001
DQGPVGRTGETGASGP[Oxi]PG	Fibrillar collagen NC1 domain-containing protein	COL1A2	18,656 ^b^	23,656 ^b^	**309,185** ^**c**^	11,533 ^a^	47.713	<0.001
AHHPDDFNPSVH	GLOBIN domain-containing protein	LOC110259958	88,515	23,406	362,462	889,540	209.499	0.549
GPIGSRGPSGPP[Oxi]GPDGNKGEP[Oxi]G	Fibrillar collagen NC1 domain-containing protein	COL1A2	1213 ^a^	3841 ^a^	**208,112** ^**c**^	17,469 ^b^	32.948	<0.001
GPRGPP[Oxi]GAVGAP[Oxi]GPQG	Fibrillar collagen NC1 domain-containing protein	COL1A2	970 ^a^	248,171 ^b^	**631,331** ^**c**^	4146 ^a^	97.820	<0.001
EQEKQNPDSEFH	UP	LOC100739741	23,441	124,249	268,476	77,106	40.512	0.131
PGQ.QKNQPMTPEAVK	UP	N/A	20,703	76,285	153,275	56,800	20.676	0.080
SDGLKHLDNLK	GLOBIN domain-containing protein	LOC100515788	1297 ^a^	**6,029,690** ^**c**^	104,465 ^ab^	831,244 ^b^	956.604	0.001
GAGGGAGGGGAGAGAGGGGAGA	Glutamate metabotropic receptor 5	GRM5	74,644 ^b^	**300,710** ^**c**^	21,625 ^a^	10,893 ^a^	44.587	<0.001
GPHEREPTAL	AMP-binding domain-containing protein	SLC27A5	331,724	7805	22,616	366,344	71.764	0.085
EPDAGDDDSKGSGQ	Ras protein specific guanine nucleotide releasing factor 2	RASGRF2	951 ^a^	**164,542** ^**d**^	26,459 ^b^	68,937 ^c^	23.835	0.001
LSDLHAHKLRVDPVN	GLOBIN domain-containing protein	LOC110259958	1955 ^a^	2686 ^a^	1123 ^a^	**194,505** ^**b**^	32.480	0.006
GPN[Dea]GDSGRP[Oxi]GEP[Oxi]GLM	Fibrillar collagen NC1 domain-containing protein	COL1A2	241,883	23,838	49,764	124,129	38.072	0.145
LANVVALTMEPK	60 kDa chaperonin	N/A	194,959	43,520	245,393	5966	47.643	0.220
GDAGPP[Oxi]GPAGPTGPP[Oxi]GPIGS	Collagen alpha-1(I) chain preproprotein	COL1A1	8915	26,500	221,936	111,099	35.314	0.060
IGENIDEKPLPT	UP	N/A	120,355	142,542	144,051	459,694	61.995	0.119
AGSPGGGAAGPGPAGGGP	Ran-binding protein 9	RANBP9	**658,925** ^**c**^	84,934 ^a^	261,216 ^b^	172,460 ^ab^	85.995	0.009
DPPKTASYPVIIQ	Rhodanese domain-containing protein	TSTD2	**237,853** ^**b**^	18,072 ^a^	40,463 ^a^	29,690 ^a^	34.637	0.001
NDNPDIPKLKPDPV	Albumin	ALB	**210,501** ^**c**^	13,125 ^a^	49,642 ^b^	52,975 ^b^	29.353	0.003
ILASCGLTDAACRLL	NACHT, LRR and PYD domains-containing protein 5	Nlrp5	63,744	285,536	8094	96,696	44.753	0.087
GIIGPLGILGP	collagen alpha-1(XXVII) chain isoform X1	COL27A1	2610 ^a^	13,191 ^a^	**1,506,056** ^**b**^	22,977 ^a^	244.419	<0.001
IGAMIGAI	ATPase	CSA50_09160	124,276 ^a^	51,483 ^a^	**2,046,297** ^**b**^	99,598 ^a^	320.834	<0.001
DSGAPIKIPVGPE	ATP synthase subunit beta	ATPB	**439,736** ^**b**^	15,127 ^a^	22,057 ^a^	66,749 ^a^	67.888	0.002
LEGTLLKPNMVT	Fructose-bisphosphate aldolase	ALDOB	**5,032,015** ^**c**^	19,118 ^a^	10,580 ^a^	214,393 ^b^	811.361	<0.001
DNPDIPKLKPDPVAL	Albumin	ALB	**164,367** ^**d**^	55,725 ^c^	27,651 ^b^	16,727 ^a^	22.199	<0.001
PGQ.QSFSDGLKHLDNLK	GLOBIN domain-containing protein	LOC100515788	9644 ^a^	14,854 ^a^	2152 ^a^	**1,049,207** ^**b**^	179.207	0.017
WDGLNPDKLYT	UP	PEBP1	99,491 ^a^	358,524 ^b^	32,772 ^a^	326,020 ^b^	57.198	0.034
SGNPNGEGLPHWP	Carboxylic ester hydrolase	APLE	19,318 ^b^	5361 ^a^	**557,870** ^**c**^	12,771 ^ab^	89.312	<0.001
PGQ.QSFSDGLKHLDNLKGTFAK	GLOBIN domain-containing protein	LOC100515788	340	530	487	791,589	142.101	0.051
GPPLRPDPSTPDFL	Dihydropyrimidinase	DPYS	**382,297** ^**c**^	12,485 ^b^	1754 ^a^	3846 ^a^	61.622	<0.001
DFQEDEQKFW	Albumin	ALB	526,201 ^b^	**1,619,475** ^**c**^	17,232 ^a^	400,233 ^b^	226.685	<0.001
GIPIPVTPKNPW	Argininosuccinate synthase	ASS1	**809,850** ^**b**^	12,991 ^a^	764 ^a^	5895 ^a^	131.502	<0.001
DQLHVDPENFRLLG	GLOBIN domain-containing protein	LOC100515788	33,561 ^b^	7863 ^a^	**603,419** ^**c**^	5475 ^a^	96.371	<0.001
AMPDIPVPLTN	Aldehyde dehydrogenase 1 family member A1	ALDH1A1	35,049 ^a^	188,598 ^a^	12,213 ^a^	**576,409** ^**b**^	92.337	0.037
DQLHVDPENFRLL	GLOBIN domain-containing protein	LOC100515788	1047 ^a^	9104 ^a^	1585 ^a^	**232,710** ^**b**^	40.397	0.035
SVGPVGPAGPI	collagen alpha-2(I) chain isoform X2	LOC101341020	**1,210,722** ^**c**^	1,126,713 ^c^	476,081 ^b^	7974 ^a^	187.098	<0.001
PGQ.QLHVDPENFRLLG	GLOBIN domain-containing protein	LOC100515788	3571 ^a^	14,016 ^a^	446 ^a^	**268,209** ^**b**^	45.742	0.025
PGQ.QLHVDPENFRLL	GLOBIN domain-containing protein	LOC100515788	4500	9124	10,757	702,733	125.698	0.058
LVLMILVL	Cytochrome b	CYB	**427,002** ^**b**^	18,871 ^a^	15,799 ^a^	582 ^a^	68.566	<0.001

Only those peptides with a concentration higher than 150,000 in at least one treatment group are shown. Abundance mean values of those peptides with significant differences (*p* < 0.05) are noted by a–d upper letters and the highest peptide value are marked in bold.

**Table 4:** Correlations between antioxidant capacity and peptide quantification obtained from alcalase, bromelain, flavourzyme, and papain treatments on pork liver.

Peptide	Protein of Origin	Gen	DPPH	ABTS	ORAC	Enzyme
SVGPVGPAGPI	Collagen alpha-2(I) chain isoform X2	LOC101341020	0.833 *			Alcalase
SP[Oxi]GPDGKTGPP[Oxi]GPAG	Collagen alpha-1(I) chain preproprotein	COL1A1	0.850 **			Bromelain
GSP[Oxi]GPSGSP[Oxi]GQRGEP[Oxi]GP	Collagen type III alpha 1 chain	COL3A1	−0.777 *		−0.715 *	Papain
GAP[Oxi]GDKGETGPSGPAGPT	Collagen alpha-1(I) chain preproprotein	COL1A1	−0.843 **			Papain
NDNPDIPKLKPDPV	Albumin	ALB		0.917 **	0.729 *	Alcalase
DNPDIPKLKPDPVAL	Albumin	ALB		0.990 **	0.788 *	Alcalase
DFQEDEQKFW	Albumin	ALB	0.836 *			Bromelain
SDGLKHLDNLK	GLOBIN domain-containing protein	LOC100515788	0.753 *			Bromelain
LVLMILVL	Cytochrome b	CYB		0.983 **	0.765 *	Alcalase
LEGTLLKPNMVT	Fructose-bisphosphate aldolase	ALDOB		0.986 **	0.761 *	Alcalase
GPPLRPDPSTPDFL	Dihydropyrimidinase	DPYS		0.991	0.776 *	Alcalase
GIPIPVTPKNPW	Argininosuccinate synthase	ASS1		0.990 **	0.770 *	Alcalase
GAGGGAGGGGAGAGAGGGGAGA	Glutamate metabotropic receptor 5	GRM5	0.916 **			Bromelain
AGSPGGGAAGPGPAGGGP	Ran-binding protein 9	RANBP9		0.875 **	0.757 *	Alcalase
DPPKTASYPVIIQ	Rhodanese domain-containing protein	TSTD2		0.966 **		Alcalase
DSGAPIKIPVGPE	ATP synthase subunit beta	ATPB		0.963 **		Alcalase
TDPDAPSRKDPKYR	UP	PEBP1	0.937 **			Bromelain

Only those correlations significant (at ** *p* < 0.01 and * *p* < 0.05) and with a correlation coefficient higher than 0.5 are shown. The FRAP test is missing because no significant correlations were obtained.

## Data Availability

The data is contained within the article.
